# Pharmacokinetics and safety of the antitumor drug everolimus in healthy Chinese subjects: a single-dose, open-label, randomized, two-sequence, two-period crossover, phase I bioequivalence study

**DOI:** 10.3389/fphar.2025.1718032

**Published:** 2025-12-18

**Authors:** Xiaodan Chen, Xiaoyi Yi, Yuting Liu, Xiaosong Wang, Yahui Peng, Qiong Wang, Ying Shuai, Hong Zhang

**Affiliations:** 1 Clinical Medicine Research Center, Jiangxi Cancer Hospital & Institute (The Second Affiliated Hospital of Nanchang Medical College), Nanchang, China; 2 Jiangxi Key Laboratory of Translational Research for Cancer, Jiangxi Clinical Research Center for Cancer, Nanchang, China

**Keywords:** bioequivalence, everolimus, healthy subject, pharmacokinetics, the antitumor drug

## Abstract

**Purpose:**

It aims to evaluate the pharmacokinetic (PK) similarity and safety of everolimus in whole blood under fasting and postprandial conditions and its generics in Chinese healthy subjects.

**Methods:**

Healthy subjects were enrolled in a single-dose, open-label, randomized, two-sequence, two-period crossover study (Clinical trial registry number: CTR20220446) under fasting and postprandial conditions. The blood concentration of everolimus was quantified by a validated and robust liquid chromatography-tandem mass spectrometry (LC-MS/MS) method.

**Results:**

All 90% CIs for the geometric mean ratios of C_max_, AUC_0-t_, and AU $C_{0-\infty }$ were within the bioequivalence range of 80.00%–125.00%. the maximum intra-individual CV% of variation were 22.76% and 18.63% under fasting and fed conditions, respectively. The gastrointestinal absorption rate was decreased by food intake: median T_max_ was 0.75 h in the fasted state, and 3.00 h after a high-fat meal. The average elimination half-life (t_1/2β_, 37.19–38.43 h) and mean residence time (MRT, 23.13–25.81 h) did not appear to be affected by food intake regardless of fasting or fed state. In this study, no serious AE (SAEs) were observed throughout the trial.

**Conclusion:**

The systemic availability of a single oral 5 mg dose of everolimus is significantly reduced by coadministration with fed compared with fasting conditions, the test formulation (*T*) and the reference formulation (*R*) were bioequivalent.

**Clinical Trial Registration:**

clinicaltrials.gov, identifier CTR20220446, (http://www.chinadrugtrials.org.cn/clinicaltrials.searchlist.dhtml).

## 1 Introduction

Everolimus, a mammalian target of rapamycin (mTOR) inhibitor, achieves triple anti-tumor effects by blocking the PI3K-AKT-mTOR signaling pathway: inhibiting tumor cell growth, reducing tumor cell nutrient metabolism, and affecting tumor angiogenesis (Menzel et al., 2023; Park et al., 2023). Everolimus was first developed by Novartis of the Swiss company, the product name was Zortress® for transplantation in the United States and Certican in Europe, and Afinitor® for cancer.

The phase III and II clinical trials of everolimus or placebo plus best supportive care in patients with metastatic renal clear cell carcinoma who had failed tyrosine kinase inhibitor (TKI) therapy have firmly established its position in subsequent therapy among patients with this characteristic (Motzer et al., 2010; Motzer et al., 2014; Motzer et al., 2016). In 2009, it was initially approved by the Food and Drug Administration (FDA) for adult patients with advanced renal cell carcinoma who had failed previous treatment with sunitinib or sorafenib. It was then successively approved for the prevention of organ rejection after kidney transplantation, tuberous sclerosis (TS), breast cancer and neuroendocrine tumors (Yao et al., 2016; Hirabatake et al., 2022; Farmaki et al., 2023). The results of the prestigious EXIST studies have revealed that everolimus can make tuberous sclerosis complex renal angiomyolipoma (TSC-AML) shrink rapidly and permanently, and so far it is the unique drug approved worldwide for the allopathic treatment of TSC-AML and tuberous sclerosis complex subventricular giant cell astrocytoma (TSC-SEGA) (Franz et al., 2013; Bissler et al., 2013; French et al., 2016). In June 2013, Afinitor® was approved by the Chinese drug regulatory authority for the treatment of late-stage renal cell carcinoma patients who had previously failed to receive sunitinib or sorafenib treatment. However, the high cost of original products has limited accessibility for many patients. Generic products may provide a viable solution to reduce patients’ financial burden and enhance the accessibility of antitumor drugs, because generic drugs usually cost less than original products.

Current publications on everolimus are more often derived from population pharmacokinetic (PPK) studies in specific populations (e.g., oncology patients) (Park et al., 2023; Tanaka et al., 2016; Combes et al., 2018; de Wit et al., 2016; Itohara et al., 2022), and have not been explored in the healthy Chinese population. The instructions for everolimus tablets show that in healthy subjects, high-fat meals reduced systemic exposure to everolimus 10 mg tablet (as measured by AUC) by 22% and the peak plasma concentration C_max_ by 54%. Light fat meals reduced AUC by 32% and C_max_ by 42%. Food, however, had no apparent effect on the post-absorption phase concentration-time profile. This data does not reflect healthy individuals in China. Everolimus is an oral mTOR inhibitor, therefore when oral drugs are administered, food-drug interactions, also known as the “food effect” affect the pharmacokinetics and efficacy of oral drug. According to the dose-dependent pharmacokinetic characteristics of everolimus in the five currently marketed generic drugs, it may be more appropriate and convenient to test it in whole blood rather than in plasma. As a result of these considerations, this study (Clinical trial registry number: CTR20220446, http://www.chinadrugtrials.org.cn/clinicaltrials.searchlist.dhtml) aims to evaluate the pharmacokinetic (PK) similarity and safety of everolimus in whole blood under fasting and postprandial conditions and its generics in Chinese healthy subjects with a randomized, single-dose, open-label, two-period crossover clinical trial.

## 2 Methods

### 2.1 Subjects

This study was conducted at the Phase I clinical trial research center of Jiangxi Cancer Hospital from April to June 2022. Eligible subjects were selected from healthy Chinese male and female volunteers aged 18–45 years, with body weight ≥50 kg, and body mass index (BMI) of 19.0∼28.0 kg/m^2^. A full medical examination was performed on all subjects including physical examinations, vital signs (oral body temperature, pulse rate, and sitting blood pressure), 12-lead ECG, and prescribed laboratory tests (routine blood, urinalysis, blood biochemical tests, coagulation function tests, Infectious diseases tests, etc.). The duration of study participation for each subject was about 33 days.

### 2.2 Study drug

The *T* formulation (everolimus tablet: 5 mg, batch number: PEMA2110001) was produced by Bo Rui Pharmaceutical (Suzhou) Co., Ltd. The *R* formulation (Afinitor®: 5 mg, batch number: SYC43) was produced by Novartis Pharma Stein AG. Both T and R were given the same batch of drug throughout the trial.

### 2.3 Ethics approval and study population

This clinical study was approved by the Medical Ethics Committee of Jiangxi Cancer Hospital with written informed consent from all subjects (IRB Approval No:2022001-YW001). The study was performed following the ethical standards for studies in humans of the Declaration of Helsinki and its amendments, the International Conference on Harmonisation Guideline for Good Clinical Practice, and the Guideline for Good Clinical Principles recommended by the National Medical Products Administration (NMPA) of China. The study reported following the Consolidated Standards of Reporting Trials (CONSORT) extension for Bioequivalence Studies (Hopewell et al., 2025). The completed checklist is provided as supplementary material.

### 2.4 Study design

This whole study was a randomized, open-label, two-sequence, two-period crossover study with a 1-week washout period. Throughout the study, each subject receives *T* or *R* is determined by a random table. Two randomization tables by SAS version 9.4 (SAS Institute Inc., Cary, NC, USA) were generated for the fasting cohort and the fed cohort through a 1:1 block randomization method. In the fasting cohort, the 28 subjects received the everolimus formulations in the sequence *T* then *R* or *R* then *T* under an overnight fast for at least 10 h; in the fed cohort, the 28 subjects received the everolimus formulations in the sequence *TR* or *RT* under the high-fat meal condition. During the trial, the unblinded pharmacist provided coded medications to the research nurse according to the study phase. The research nurse subsequently administered these medications to the participants.

The instructions for Afinitor® indicate that the C_max_ is about 1–2 h after oral administration of 5–70 mg in patients with advanced solid tumors. The t_1/2β_ of everolimus is about 30 h, which can meet the requirement of sampling time of 3–5 t_1/2β_ or more for 96 h, and the AUC_0-t_ of everolimus in fed condition can cover 80% of AU $C_{0-\infty }$ . Venous blood samples (3 mL) were collected from an intravenous indwelling catheter before the dose and at 0.25, 0.5, 0.75, 1, 1.5, 2, 3, 4, 6, 8, 12, 24, 36, 48, 72, and 96 h after the 5 mg dose in the fasting cohort, and at 0 (pre-dosing), 0.5, 0.75, 1, 1.5, 2, 2.5, 3, 4, 5, 6, 8, 12, 24, 36, 48, 72, and 96 h after the dose in the fed cohort of the study. All participating subjects were under medical supervision by a physician throughout the study. After sample collection, the blood was stored directly in the −80 °C refrigerator until analyzed by LC-MS/MS.

### 2.5 Safety assessment

Safety assessments included physical examinations, vital signs, 12-lead ECGs, prescribed laboratory tests, and AE monitoring.

### 2.6 Determination of blood concentrations

In this study, the concentration of everolimus in blood was determined by a validated LC-MS/MS method. A deuterium-labeled analytical internal standard (IS, [^13^C_2_,^2^H_4_]- everolimus) was used for the quantitative determination of everolimus. To ensure the reliability of the analytical method for the determination, validation of this analytical method was performed to evaluate its selectivity, linearity, precision, accuracy, matrix effect, extraction recovery, and stability in matrix samples according to ICH M10 criteria.

A protein precipitation method was used to extract everolimus from human blood samples: An aliquot (50 μL) of IS (10.0 ng/mL in 50% [v/v] acetonitrile) was added to 50 μL of blood, then 200 μL of acetonitrile was added into each sample and mixed well (vortex 2,500 rpm × 3 min). After centrifuging the mixture at 14,000 rpm × 5 min at 4 °C, the supernatant was transferred into auto-sampler vials, and 20 μL was injected into the LC-MS/MS system for analysis. Chromatographic conditions were described as follows: Quantitation was achieved on a TRIPLE QUAD 5500 mass spectrometer (Applied Biosystems Sciex, Foster, CA, USA) in positive-ion mode with electrospray ionization (ESI) in multiple-reaction-monitoring (MRM) mode (m/z 975.600/908.600 for everolimus, m/z 981.600/914.600 for IS). The analyte and IS were separated on an Agela Technologies Venusil XBP C8 (2.1 × 50 mm, 5 μm) using a gradient elution program with a flow rate of 0.4 mL/min. The mobile phases A was methanol, and mobile phases B was 0.1% ammonium formate in water.

The lower limit of quantitation (LLOQ) of everolimus was 0.100 ng/mL with a dynamic range of 0.100–100 ng/mL. Quality control (QC) samples (Low QC, 0.300 ng/mL; Geometric Mean QC, 3.00 ng/mL; Medium QC, 30.0 ng/mL; High QC, 80.0 ng/mL; Dilution QC, 600 ng/mL) were analyzed to assess the accuracy and precision of the method. All QC samples are evenly distributed among the samples to be analyzed. The retention times of everolimus and IS were about 2.56 min, respectively. The missing pharmacokinetic concentration data were not imputed.

### 2.7 Pharmacokinetic analysis

Phoenix WinNonlin 8.2 (Pharsight Corp, Mountain View, CA, USA) and SAS software version 9.4 (SAS Institute, Cary, NC, USA) were used for statistical analysis in this study. Phoenix WinNonlin software was used to estimate and analyze the PK parameters of the non-compartmental model in the blood concentration data, and the Primary PK parameters (C_max,_ AUC_0-t_, and AU $C_{0-\infty }$ ) and the secondary PK parameters (T_max_, MRT, t_1/2_, and λ_z_). Sensitivity analysis was performed using Phoenix WinNonlin software.

### 2.8 Bioequivalence assessment

The average bioequivalence (ABE) method was used to evaluate the bioequivalence of the *T* and *R* formulations. When the 90% confidence interval (CI) of the geometric mean ratio of C_max_, AUC_0-t_, and AU $C_{0-\infty }$ between the *T* and *R* fall within the range of 80%–125% in this fasting and fed study, it can be determined that the two formulations are bioequivalent. SAS software was used for statistical analysis of data, and differences were considered statistically significant at p < 0.05.

## 3 Results

### 3.1 Volunteers characteristics

**FIGURE 1:**
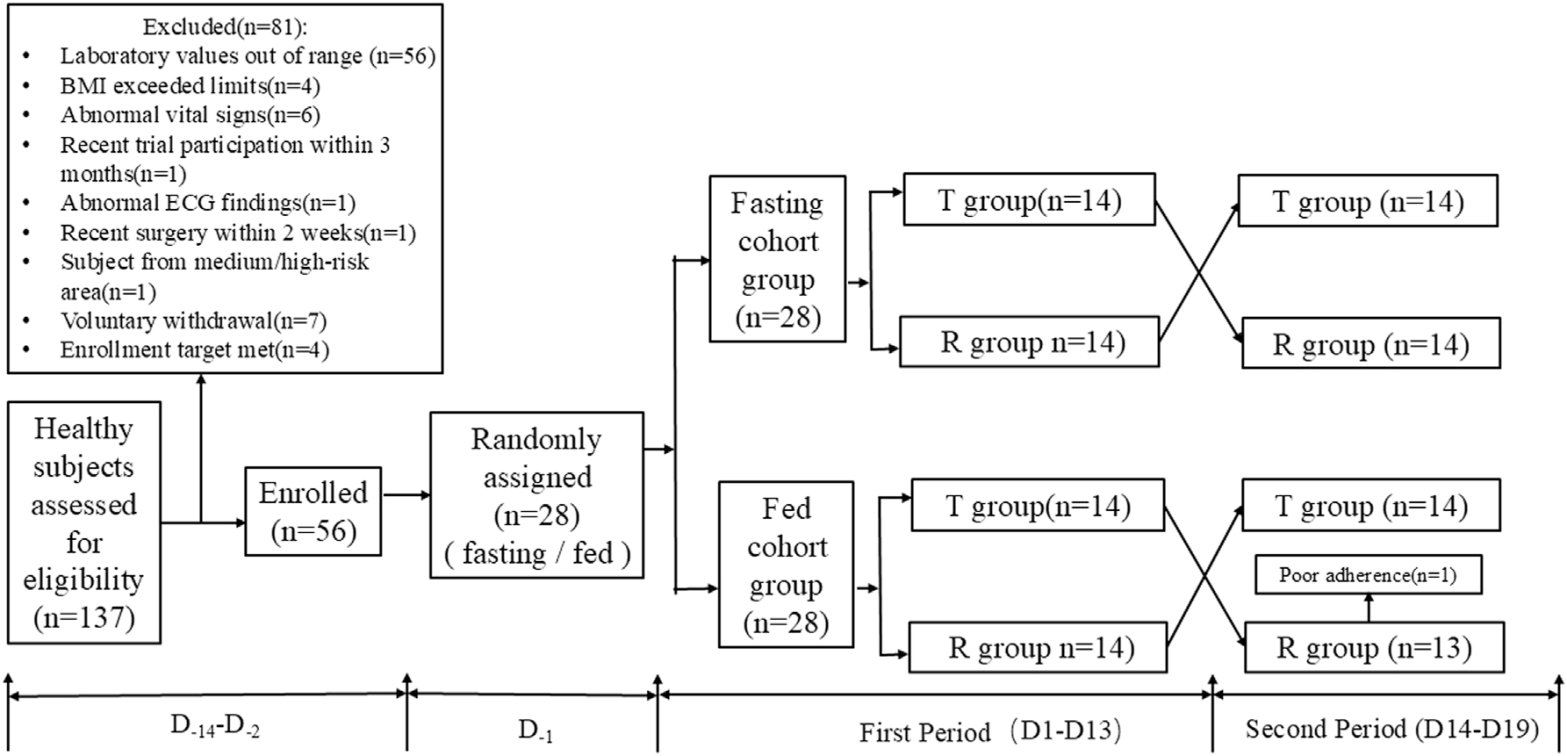
The study flow chart under fasting and fed conditions.

Twenty-eight subjects were included in each of the fasting and fed cohort groups (fasting: male: female = 22:6; postprandial: male: female = 25:3), with mean values of age, weight, and BMI of 27.57 ± 8.61 years, 63.95 ± 10.44 kg, and 22.51 ± 2.59 kg/m^2^ in the former and 26.14 ± 7.23 years, 63.45 ± 8.72 kg, and 22.17 ± 2.22 kg/m^2^ in the latter, respectively. Table 1 shows the demographic factors and laboratory tests of the two groups. All subjects received a single 5 mg oral dose of a test everolimus tablet (BoRui Pharmaceutical (Suzhou) Co., Ltd.) and the reference product (Afinitor®, Novartis Pharma Stein AG) under fasting or fed conditions. In the fed cohort group, subject Y025 was discontinued after failing to swallow the study drug whole in period 2, All other 27 participants completed the study in full compliance with the trial protocol (Figure 1).

**TABLE 1:** Demographic characteristics of the subjects.

Parameters^a^	Under fasting condition (N = 28)	Under fed condition (N = 28)
Age (years)	27.57 ± 8.61	26.14 ± 7.23
Sex (n %)		
Male	22 (78.57%)	25 (89.28)
Female	6 (21.43%)	3 (10.72)
Height (cm)	168.38 ± 9.75.4	169.04 ± 7.81
Weight (kg)	63.95 ± 10.44	63.45 ± 8.72
BMI (kg/m^2^)	22.51 ± 2.59	22.17 ± 2.22

^a^ Values are presented as mean ± SD, except for sex, which is shown as n (%).

### 3.2 Method validation

The bioanalytical method validation results are briefly described as follows: No interfering peaks were observed near the retention times of everolimus and IS for all samples, The intra-batch and inter-batch accuracy and precision were assessed by six runs analyzed at four concentration levels of QCs. Including the LLOQ, the intra-batch accuracy deviation (%DEV) was −3.94%∼7.50%, and the precision (coefficient of variation, %CV) ranged from 2.81%∼9.16%. For Inter-batch accuracy and precision, the %DEV was −1.86%∼6.77%, and the %CV was 4.92%∼14.87% (Table 2).

**TABLE 2:** Intra-batch and Inter-batch precision and accuracy of the LC-MS/MS method for determining everolimus concentration in human blood.

Concentration Added (ng/mL)	Intra-batch (N = 6)			Intre-batch (N = 6)		
	Mean ± SD (ng/mL)	Accuracy (%DEV)	Precision (CV%)	Mean ± SD (ng/mL)	Accuracy (%DEV)	Precision (CV%)
0.100	0.102 ± 0.01	2.42	9.16	0.107 ± 0.02	6.77	14.87
0.300	0.288 ± 0.01	−3.94	4.86	0.312 ± 0.03	3.92	9.46
3.00	3.23 ± 0.09	7.50	2.81	3.18 ± 0.16	5.87	5.06
30.0	31.1 ± 1.03	3.56	3.32	30.4 ± 1.59	1.44	5.22
80.0	78.3 ± 2.91	−2.17	3.72	78.5 ± 3.87	−1.86	4.92

The matrix effect was assessed by preparing LQC, MQC, and HQC using six different sources of normal blank matrix and three different sources of haemolysed matrix with three replicates. The average normalized matrix factor of everolimus was 1.03, 1.00, and 0.99, respectively, and the overall %CV was 0.71∼4.69%, showing that the ion inhibition or enhancement in human blood can be negligible at different matrices. By calculating the peak area ratio of LQC, MQC, and HQC, the extraction recoveries of everolimus in blood were 95.57%, 95.14%, and 91.78%, respectively, and the overall %CV was 2.97%; the extraction recovery of IS was 99.97%, and the %CV was 2.61% (Table 3).

**TABLE 3:** Matrix factor and recovery of everolimus and IS.

Concentration Added (ng/mL)	Matrix factor^a^ (N = 27^c^)		Recovery^b^ (N = 18^d^)		
	Matrix factor	%CV	Analyte (%)	IS (%)	%CV
0.300	1.03	4.69	95.57	99.97	2.61
30.0	1.00	1.03	95.14		
80.0	0.99	0.71	91.78		

^a^Matrix factor (Non − Normalized) = Average peak area response in the presence of matrixions (Matrix Factor Blanks)/Average peak area response in the absence of matrixions (Neat Samples).

^b^Recovery = Average peak area response in the presence of Pre - extraction/Average peak area response in the presence of Post - extraction.

^c^Each QC, for matrix effect was assessed by six different sources of normal blank matrix with three replicates and three different sources of haemolysed matrix with three replicates.

^d^Each QC, for recovery was assessed by six different sources of normal blank matrix with three replicates.

The testing included the stability of whole blood at room temperature for 19 h, frozen-thawed cycles five times, processed samples in the autosampler (4 °C) for 212 h, and the long-term stability at −80 °C condition for 44 days, the results of matrix stability were all within the acceptable criteria.

**FIGURE 2:**
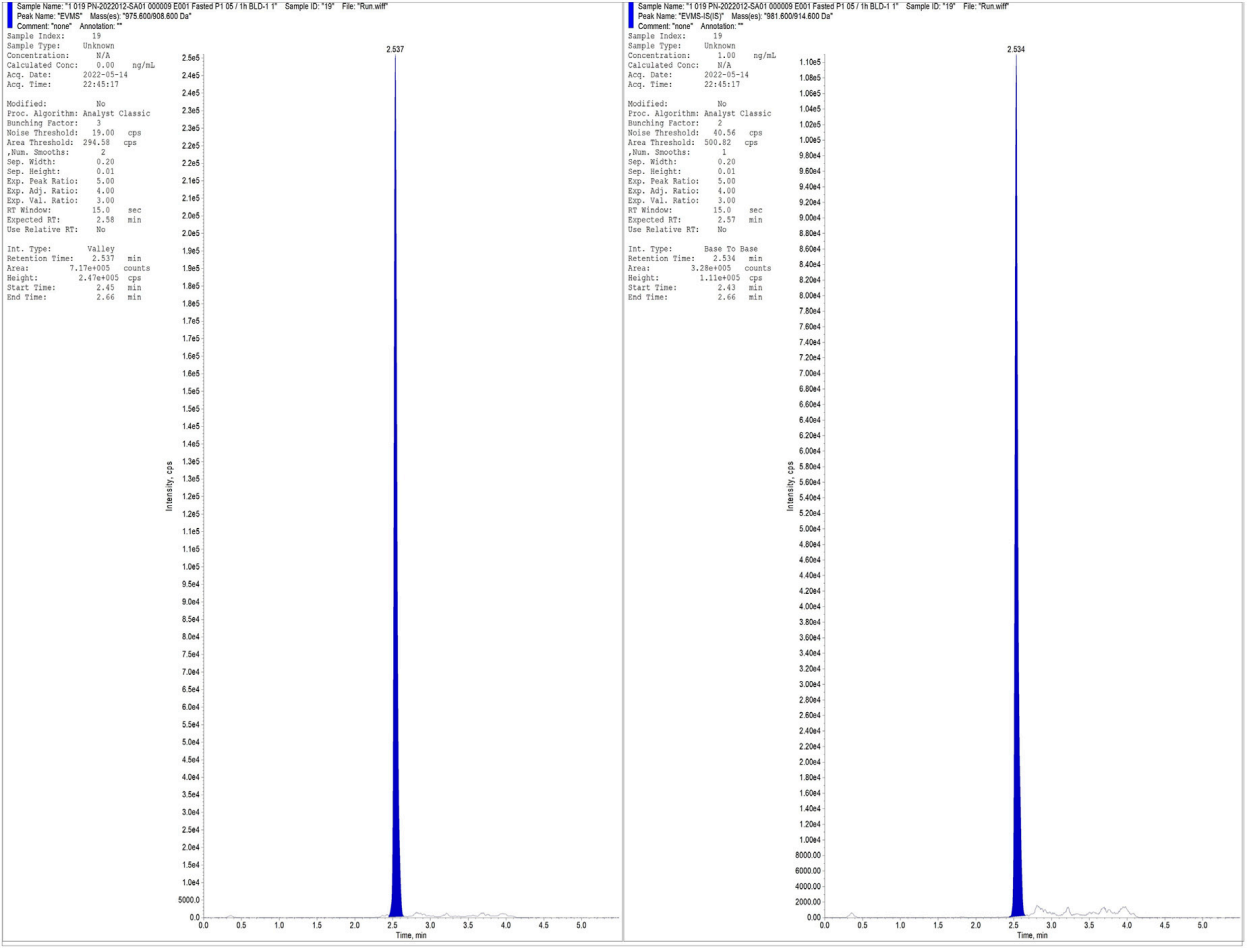
Typical chromatogram of Everolimus and IS for E001 subject after administration.

In summary, this analytical method exhibits high sensitivity, simple sample processing steps (using protein precipitation method), no matrix effect, good recovery, and a short run time (2.56 min) (Figure 2), which is particularly well-suited for routine analysis of a large number of samples.

### 3.3 Pharmacokinetics

**FIGURE 3:**
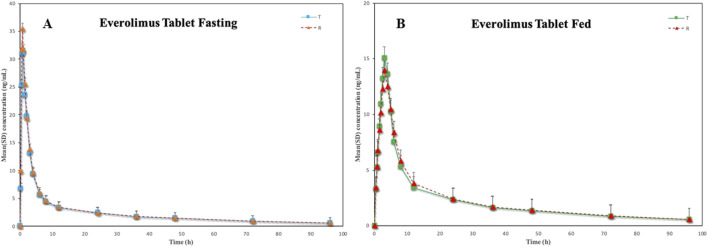
Mean (±SD) blood concentration-time curves of everolimus following single-dose oral administration under fasting (**(A)**, N = 28) and fed (**(B)**, N = 28) conditions in healthy Chinese volunteers.

Under fasting cohort, the average relative bioavailability of F_0-t_ and $F_{0-\infty }$ was (99.83 ± 20.11) % and (98.11 ± 18.78) %, respectively; under fed cohort, F_0-t_ and $F_{0-\infty }$ were (98.79 ± 12.30) % and (98.14 ± 11.80) %, respectively. The concentration-time curves of the two groups after receiving a single oral 5 mg everolimus tablet are described in Figure 3, and the main pharmacokinetic parameters are illustrated in Table 4. Under fasting cohort, the intra-subject CV% for C_max_ was 33.21% for the test (T) formulation and 34.26% for the reference (R) formulation, while the intra-subject CV% for AUC_0-t_ were 35.25% (T) and 21.92% (R),These results showed considerable inter-individual variability in the absorption of everolimus; In contrast, under fed cohort, the intra-subject CV% for C_max_ of the *T* and *R* formulations were 25.61% and 29.91%, respectively, and the intra-subject CV% for AUC_0-t_ were 15.62% and19.98%. Food intake may moderate inter-individual pharmacokinetic differences. Based on the median and range of T_max_, suggesting that food intake significantly delayed drug absorption, which aligns with the expected effect of a high-fat diet on gastrointestinal motility. For R formulation, the gastrointestinal absorption rate was decreased by food intake: median Tmax was 0.75 h in the fasted state, and 3.00 h after a high-fat meal. The average elimination half-life (t1/2β, 37.19–38.43 h) and mean residence time (MRT, 23.13–25.81 h) did not appear to be affected by food intake regardless of fasting or fed state. In addition, the results of the Wilcoxon signed-rank test did not exhibit any statistical significance in the T_max_ between the two formulations under fasting or fed condition (*P* > 0.05).

**TABLE 4:** Pharmacokinetic properties of T and R formulations of everolimus 5-Mg tablets after single-dose administration in healthy Chinese volunteers under fasting and fed Conditions (Mean ± SD (intra CV%)).

Parameters	Under fasting condition		Under fed condition	
	*T* Formulation	*R* Formulation	*T* Formulation	*R* Formulation
T_max_ (h)^b^	0.75 (0.50, 4.00) (75.61)	0.75 (0.50, 3.00) (58.20)	2.75 (0.75, 4.00) (40.28)	3.00 (0.50, 6.00) (44.88)
C_max_ (ng/mL)	35.38 ± 11.75 (33.21)	39.04 ± 13.38 (34.26)	18.94 ± 4.85 (25.61)	18.34 ± 5.48 (29.91)
AUC_0-t_ (ng·h/mL)	234.62 ± 82.70 (35.25)	245.44 ± 53.81 (21.92)	212.36 ± 33.16 (15.62)	217.86 ± 43.52 (19.98)
AU $C_{0-\infty }$ (ng·h/mL)	265.73 ± 96.28 (36.23)	278.28 ± 61.99 (22.28)	243.34 ± 39.18 (16.10)	251.30 ± 51.65 (20.55)
AUC_%Extrap_ (%)	13.36 ± 10.64 (79.69)	11.66 ± 3.35 (28.70)	12.60 ± 2.98 (23.68)	13.15 ± 3.07 (23.32)
t_1/2_ (h)	35.90 ± 7.13 (19.87)	37.19 ± 6.74 (18.12)	37.22 ± 4.79 (12.86)	38.43 ± 5.23 (13.60)
λ_z_ (h^-1^)	0.02 ± 0.01 (25.09)	0.02 ± 0.00 (18.24)	0.02 ± 0.00 (11.14)	0.02 ± 0.00 (13.07)
MRT_0-t_ (h)	22.88 ± 3.55 (15.52)	23.13 ± 1.83 (7.92)	25.60 ± 1.88 (7.33)	25.81 ± 1.74 (6.76)

^a^Tmax is represented by the median [minimum, maximum].

### 3.4 Bioequivalence

The average bioequivalence (ABE) method was adopted in this study. A total of 28 healthy subjects completed the fasting test, the results showed that the 90% CI of C_max_, AUC_0-t_, and AU $C_{0-\infty }$ for the geometric mean ratio (GMR) of the *T* and *R* formulations were 95.29% (85.81–105.80%), 97.46% (91.39–103.93%), and 96.85% (90.94–103.15%) (Table 5), the maximum intra-individual CV% (intra-CV%) of *T* and *R* formulations was 22.76% (C_max_) under fasting condition. A total of 28 healthy subjects completed the fed test, the results revealed that the 90% CI of C_max_, AUC_0-t_, and AU $C_{0-\infty }$ for the GMR of the *T* and *R* were 104.13% (95.61–113.41%), 97.85% (94.13–101.72%), and 97.40% (93.60–101.35%) (Table 6), the maximum intra-CV% was 18.63% (C_max_). All parameters were within the bioequivalence range of 80.00%–125.00%.

**TABLE 5:** Bioequivalence evaluation of *T* and *R* formulations of everolimus 5-Mg tablets in healthy volunteers under fasting condition.

Parameters	Geometric mean			90%CI (Lower ∼ Upper) (%)	Power (%)	Intra-CV (%)
	*T*	*R*	(T/R)%			
C_max_ (ng/mL)	30.91	29.45	95.29	85.81∼105.80	86.70	22.76
AUC_0-t_ (ng·h/mL)	203.81	198.63	97.46	91.39∼103.93	99.97	13.87
AU $C_{0-\infty }$ (ng·h/mL)	273.65	265.04	96.85	90.94∼103.15	99.97	13.36

**TABLE 6:** Bioequivalence evaluation of *T* and *R* formulations of everolimus 5-Mg tablets in healthy volunteers under fed condition.

Parameters	Geometric mean			90%CI (Lower ∼ Upper) (%)	Power (%)	Intra-CV (%)
	T	R	(T/R)%			
C_max_ (ng/mL)	17.65	18.38	104.13	95.61∼113.41	97.15	18.63
AUC_0-t_ (ng·h/mL)	214.58	209.97	97.85	94.13∼101.72	>99.99	8.38
AU $C_{0-\infty }$ (ng·h/mL)	246.47	240.05	97.40	93.60∼101.35	>99.99	8.43

### 3.5 Safety

A total of 22 AEs occurred in 15 (53.57%) subjects in the fasting cohort, including 14 AEs in 10 (35.71%) subjects under the *T* and 8 AEs in 5 (17.86%) subjects under the *R*. The AEs related to the study drug was 18 AEs in 13 (46.43%) subjects (Table 7); A total of 17 AEs occurred in 13 (46.43%) subjects in the fed cohort, including 10 AEs in 7 (25.00%) subjects under the *T* and 7 AEs in 6 (21.43%) subjects under the *R*. The AEs related to the study drug was 16 AEs in 12 (42.86%) subjects (Table 7). All AEs were mild, and none led to subject withdrawal. All AEs were followed until resolution, catagorising as “improved”, “recovered”, or “unknown”.

**TABLE 7:** Summary of adverse events (SS) of *T* and *R* formulations of everolimus 5-Mg tablets after oral administration under fasting and fed conditions in healthy Chinese volunteers.

Projects	Fasting study		Fed study	
	*T* (N = 28)	*R* (N = 28)	*T* (N = 28)	*R* (N = 28)
At least one adverse event occurred	10 (35.71%)	5 (17.86%)	7 (25.00%)	6 (21.43%)
Related to research drugs	8 (28.57%)	5 (17.86%)	7 (25.00%)	5 (17.86%)
SAE	0 (0.00%)	0 (0.00%)	0 (0.00%)	0 (0.00%)
Adverse events leading to study discontinuation	0 (0.00%)	0 (0.00%)	0 (0.00%)	0 (0.00%)

## 4 Discussion

Due to the high cytotoxicity of anti-tumor drugs, in order to truly reflect the safety and effectiveness of drugs in patients and avoid unnecessary damage to healthy subjects, cancer patients are generally selected for human studies due to ethical requirements. However, for some non-cytotoxic drugs, such as hormones, tyrosine kinase inhibitors - due to their mild toxicity, studies based on healthy volunteers can be considered to obtain relatively accurate metabolic characteristics of drugs *in vivo* provided that subject safety is fully ensured. Healthy adult volunteers were identified for the study in accordance with the FDA’s Draft Guidance on Everolimus (U.S. Food and Drug Administration, 2018), thus healthy subjects were enrolled to complete this clinical trial. Presently, no other relevant literature has reported the exploration of the pharmacokinetic parameters of everolimus in healthy humans. The pharmacokinetic data of the original drug showed that Chinese solid tumor patients (n = 24) reached peak blood drug concentrations 2–3 h after receiving 5 mg/day and 10 mg/day, which was consistent with the Tmax data of healthy subjects in our experiment, indicating that clinical trials using healthy subjects can accurately reflect real-world pharmacokinetic parameters.

The concentration of the parent drug in whole blood was determined, which is a distinguishing feature of everolimus compared to the concentration monitoring of other drugs. Everolimus exhibited dose-dependent pharmacokinetic characteristics ranging from 5 to 5,000 ng/mL. Upon oral administration, approximately 75% of the gastrointestinally absorbed penetrated the erythrocytes, while the plasma drug concentration accounted for about 20% of the whole blood concentration. Despite the fact that six major metabolites, including three monohydroxylated metabolites, two hydrolysed ring-opening products, and one phosphatidylcholine conjugate were detectable in human blood, they were considered negligible, as their activity was about 1% of the original. Therefore, the concentration of the parent drug in whole blood was determined to evaluate bioequivalence.

Under both fasting and fed conditions, the 90% confidence intervals for the geometric mean ratios of the primary pharmacokinetic parameters between the test and reference formulations fell entirely within the predefined bioequivalence range. The intra-subject coefficients of variation for both formulations were within acceptable limits under both prandial states, and the mean relative bioavailability was comparable. Furthermore, statistical analysis of T_max_ showed no significant differences between the two formulations under either fasting or fed conditions (P > 0.05). As such, the two products were bioequivalent under both fasting and fed conditions. Furthermore, the AUC%_Extrap_ values were well below 20% (Fasting T: 13.36%, R: 11.66%; Fed T: 12.60%, R: 13.15%), confirming that the blood sampling schedule was appropriately designed to reliably estimate total drug exposure from time zero to infinity (AU $C_{0-\infty }$ ). the elimination half-life (t_1/2_, approximately 35–38 h) and terminal elimination rate constant (λ_z_) were highly consistent. This demonstrates that the elimination processes of the test and reference formulations were fundamentally equivalent, further supporting the conclusion of bioequivalence between the two products.

Sensitivity analysis was performed in this study. In the fasting cohort, because the AUC_%Extrap_ of E019 and E020 subjects in the first period were both greater than 20%, the AU $C_{0-\infty }$ analysis set did not include them. The sensitivity analysis of AU $C_{0-\infty }$ in 28 subjects was carried out, Phoenix WinNonlin 8.2 software was used to analyze BES, and the results showed that the 90% CI of *T/R* GMR of AU $C_{0-\infty }$ was 91.05–103.51%, and the *P* values of AU $C_{0-\infty }$ were all <0.05. In the fed cohort, because the AUC_%Extrap_ of Y007 subjects in the first and second periods were both greater than 20%, the AU $C_{0-\infty }$ analysis set did not include them. The sensitivity analysis of AU $C_{0-\infty }$ after inclusion showed that the 90% CI of *T/R* GMR of AU $C_{0-\infty }$ was 93.65∼101.11%. Both unilateral *t-tests* showed that *P* values of AU $C_{0-\infty }$ were <0.05. The fact that both *T* and *R* formulations are bioequivalent was demonstrated in both parts of the sensitivity analysis study.

## 5 Conclusion

In the study, it is demonstrated that it is safe for healthy subjects to participate in bioequivalence studies of antitumor drugs. A simple and high-throughput LC-MS/MS method with satisfactory accuracy, precision, and sensitivity was developed for the detection of everolimus in human blood. The validated method allows the determination of everolimus concentrations in the range of 0.100–100 ng/mL, making it is particularly applicable for routine analysis a large sample volumes.

The systemic availability of a single oral 5 mg dose of everolimus was significantly reduced when coadministration with food compared to under fasting conditions. Moreover, the trial concluded that the *T* formulation (5 mg tablet) was bioequivalent to the *R* formulation under both fasting and fed conditions.

## Data Availability

The raw data supporting the conclusions of this article will be made available by the authors, without undue reservation.
